# Progress in Bacteriology

**Published:** 1901-10-12

**Authors:** 


### Progress in Bacteriology.

(*Continued from page* 399.)

**Ozæna.**—Terez[9] contributes a second examination of the bacteriology of this disease. He thinks that the claims of the bacillus mucosus advocated by Löwenfeldt and Abel may be disregarded, as this organism never gives rise to fœtor in cultures and has never produced ozæna when inoculated into animals. From an examination of a further series of cases he is of opinion that the cocco-bacillus fœtidus ozæna described by him in 1899[10] is the causative factor. The characteristics of this organism are its polymorphism, and the characteristic smell evolved from the cultures. The organism may be found as an oval coccus, or as a small or large bacillus. It is immobile, stains with all the aniiine dyes, but not with Gram's method. In broth it forms a cloud and a deposit, but no film, and on solid media, thick shining, semi-transparent colonies appear, and the organism shows a tendency to run in sinuous chains. It does not liquefy gelatine or ferment lactose or milk. It forms indol in broth, and decomposes urea. This cocco-bacillus when injected into rabbits leads to ozæna and atrophy of the turbinates and nasal mucous membranes. Perez gives a list of twelve instances in which multiple cases of ozæna appeared in a household, and argues from these that the disease is contagious. He also asserts that the cocco-bacillus is present in the nasal passages of dogs suffering from diseased conditions, and is of opinion that the disease is frequently transmitted to man from this source. In order to obtain the characteristic smell from cultures they must be securely sealed during incubation. Liquid media give a more powerful smell than solid.

**Cancer.**—Gaylord[11] reviews the results of various observers with regard to the parasitology of cancer. Busser, in 1893, described a form of yeast cell associated with malignant tumours. Sanfelice succeeded in producing local tumours and secondary lymphatic enlargement by means of inoculation with a species of saccharomyces. By passage through a series of animals the virulence of these tumours was increased. Russell's fuchsin bodies, he thinks, were varieties of yeast cells, and the same opinion as to the causation of cancer was supported by Plimmer and by Roncali. Pfeiffer held that the organism belonged to the protozoa, and in this view he was supported by Munk. Gaylord's own researches were conducted on unstained tissues, the cancer bodies appearing as small masses resembling fat, but with a definite capsule, and not giving the osmic acid test. Injected into the peritoneum of animals they excited a peritonitis, and could be recovered in large numbers from the effusion. Cancer bodies were extremely difficult to cultivate. Sixty different media had been employed and only once had yeast been present in pure culture. One successful inoculation was recorded, the fluid from the peritoneum in a case of abdominal cancer being injected into the jugular vein of a guinea-pig gave rise to a primary adeno-carcinoma of the lung. Cancer bodies were to be found in all malignant growths. They have a peculiar mobility, and go through several stages of development, and multiply by sporulation. In Gaylord's opinion they should be classified with the protozoa.

9 An. de l'Inst. Past., Aug. 25, 1901. 10 Ibid, Dec. 25, 1899.

11 Jour. Amer. Med. Assoc., April 16, 1901.

